# Comparison of the effect of autoclaved and non-autoclaved live soil exposure on the mouse immune system: Effect of soil exposure on immune system

**DOI:** 10.1186/s12865-023-00565-0

**Published:** 2023-09-09

**Authors:** Laura Kummola, Martín I. González-Rodríguez, Pertti Marnila, Noora Nurminen, Tanja Salomaa, Lotta Hiihtola, Iida Mäkelä, Olli H. Laitinen, Heikki Hyöty, Aki Sinkkonen, Ilkka S. Junttila

**Affiliations:** 1 Faculty of Medicine and Health Technology, Tampere University, Tampere, 33014 Finland; 2 Fimlab Laboratories, Arvo-Building, Rm F326, Arvo Ylpön katu 34, Tampere, 33520 Finland; 3 Natural Resources Institute Finland (Luke), Jokioinen, Finland; 4 Northern Finland Laboratory Centre (NordLab), Oulu, 90220 Finland; 5 Research Unit of Biomedicine, University of Oulu, Oulu, 90570 Finland

## Abstract

**Background:**

. Lack of exposure to the natural microbial diversity of the environment has been linked to dysregulation of the immune system and numerous noncommunicable diseases, such as allergies and autoimmune disorders. Our previous studies suggest that contact with soil material, rich in naturally occurring microbes, could have a beneficial immunoregulatory impact on the immune system in mice and humans. However, differences in the immunomodulatory properties of autoclaved, sterile soil material and non-autoclaved, live soil material have not been compared earlier.

**Results:**

. In this study, we exposed C57BL/6 mice to autoclaved and live soil powders that had the same rich microbiota before autoclaving. We studied the effect of the soil powders on the mouse immune system by analyzing different immune cell populations, gene expression in the gut, mesenteric lymph nodes and lung, and serum cytokines. Both autoclaved and live soil exposure were associated with changes in the immune system. The exposure to autoclaved soil resulted in higher levels of *Rorγt*, *Inos* and *Foxp3* expression in the colon. The exposure to live soil was associated with elevated IFN-γ concentration in the serum. In the mesenteric lymph node, exposure to live soil reduced *Gata3* and *Foxp3* expression, increased the percentage of CD8 + T cells and the expression of activation marker CD80 in XCR1^+^SIRPα^−^ migratory conventional dendritic cell 1 subset.

**Conclusions:**

. Our results indicate that exposure to the live and autoclaved soil powders is not toxic for mice. Exposure to live soil powder slightly skews the immune system towards type 1 direction which might be beneficial for inhibiting type 2-related inflammation. Further studies are warranted to quantify the impact of this exposure in experimental type 2 inflammation.

**Supplementary Information:**

The online version contains supplementary material available at 10.1186/s12865-023-00565-0.

## Background

The biodiversity hypothesis suggests that decreased interaction with microbes can lead to an imbalance in the human microbiota (referred to as dysbiosis), which in turn will contribute to the development of immune-mediated diseases including asthma, allergies, type 1 diabetes, inflammatory bowel disease, rheumatoid arthritis, and even neurological disorders such as Alzheimer and Parkinson’s disease as well as metainflammation associated hypertension, cardiovascular diseases and type 2 diabetes [1–8]. Increasing evidence has linked the high hygiene standards of modern lifestyle and the loss of biodiversity in urbanized living environments to a decrease in the richness of indoor microbiota [9, 10] and commensal microbiota in the human skin and gut [11–15]. It is known that exposure to nature-derived microorganisms in early life is associated with the modulation of the immune system: Contact with agricultural land, living on farms, green space around home and housing a pet have been associated with the below-average risk of immune dysfunction [16–22]. Furthermore, it has been shown that people who suffer from immune-mediated diseases have lower diversity of commensal bacteria [5, 23–25].

To counterbalance this effect, different interventions have been tested to improve microbial diversity and reduce the risk of inflammatory disorders. Typically, studies have focused on probiotics, prebiotics, postbiotics and fecal transplants, and while, for instance, in the treatment of *Clostridium difficile* infection fecal transplantation treatment has proven successful [26], often attempts to address dysbiosis and immune disorders have shown mixed results [27–32]. Most methods rely on one or a few strains of bacteria while little attention has been given to exposure to natural microbial biodiversity as an immunomodulatory and therapeutic method [33].

We have previously utilized a novel approach of bringing humans and mice into contact with microbially rich plant- and soil-based material. In humans, exposure to this material increased commensal microbial diversity, which was linked to higher levels of anti-inflammatory cytokines TGF-β and IL-10 in plasma, and increased IL-10/IL-17 ratio and proportion of regulatory T cells [34–37]. The shift in the microbiota persisted for the duration of a 2-year study [38]. In mice, autoclaved forest-derived soil powder reduced the level of IL-21 and IL-17 F in serums as well as decreased the release of pro-inflammatory signals, such as IL-1β, IL-5, IL-6, IL-13 and TNF, in activated splenocytes [39]. Similar results, demonstrating an immunoregulatory effect, were also reported in a mouse soil exposure study by Ottman et al. [40].

Here, we compared the effects of autoclaved and non-autoclaved, live soil powder on the mouse immune system, to understand if soil-derived material conveys immunoregulatory signals, and to test whether the exposure to the materials is toxic for mice. To this end, we analyzed different lymphoid and myeloid subsets in the mouse spleen and gut-draining mesenteric lymph nodes (mLN) by flow cytometry and studied the expression of important immune-related genes in different organs by qPCR. We also determined the concentration of different inflammatory and anti-inflammatory cytokines in the mouse serum. To our knowledge, this is the first study to analyze how sterile and live soil materials compare as immunomodulatory tools.

## Results

### Cytokine profile in Serums

**Fig. 1:**
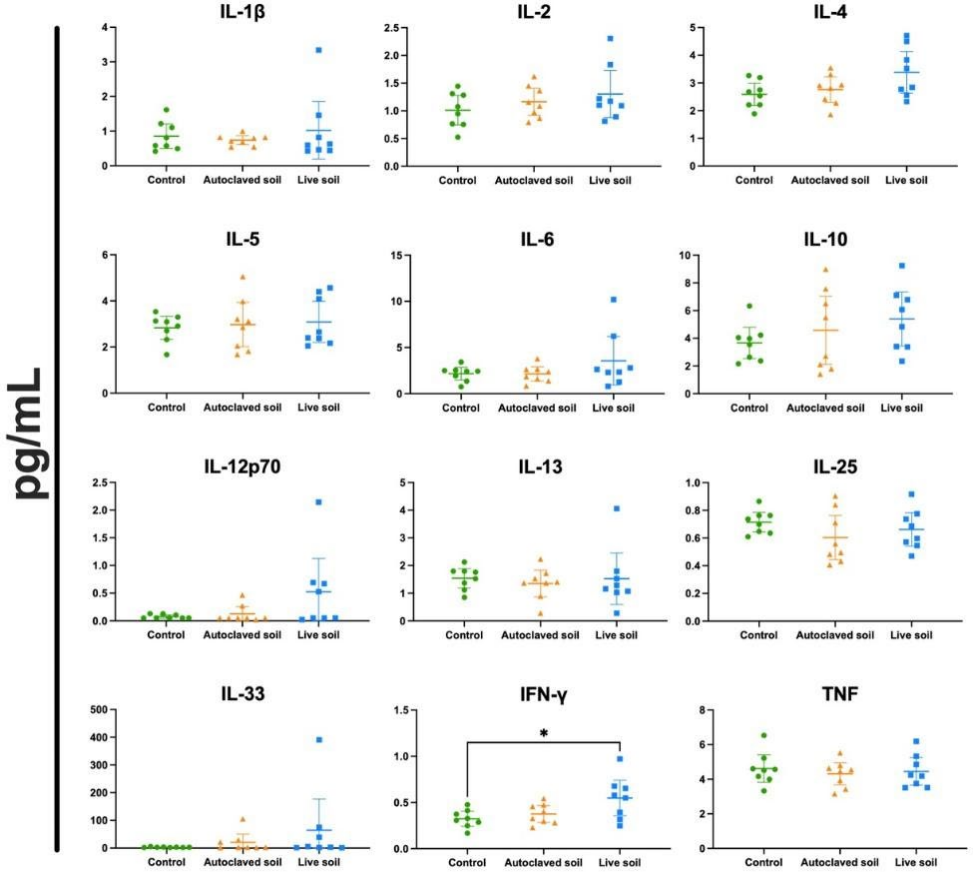
**Exposure to live soil increases serum IFN-γ concentration. Sera from exposed animals were collected at day 21 and analyzed for cytokine concentrations using U-Plex from Meso Scale Discovery**. Serum concentrations (pg/mL) are shown. Data are shown as scatter plots with mean and 95% CI (n = 8 per group, from two independent exposure experiments). Each symbol represents an individual mouse, lines indicate the mean. The data was analyzed with One-way ANOVA with Tukey´s multiple comparison test, *p < 0.05

Exposure to soil powder has been linked to changes in serum cytokine concentrations both in humans and mice [34, 35, 37, 39]. We compared the effect of the autoclaved soil powder with the live soil powder preparate in wild type C57BL/6 mice along with a control group that was maintained in clean bedding. Mice in two groups were subjected to the treatments for 1 h, 5 days a week, for three weeks, while the third i.e.control group was not in soil contact. We found an increased INF-γ in serums from animals exposed to the non-autoclaved live powder compared to the control group (p = 0.0256, Fig. 1) but the autoclaved material did not induce IFN-g. Other significant differences in serum cytokines were not observed. The measured cytokines included IL-10, IL-12p70, IL-13, IL-6, IL-25, IL-1β, IL-2, IL-4, IL-5, TNF and IL-33 (Fig. 1). Additionally, IL-17 F, IL-21 and IL-31 were measured but their levels were not detectable.

### Myeloid immune profile in mLN and spleen

**Fig. 2:**
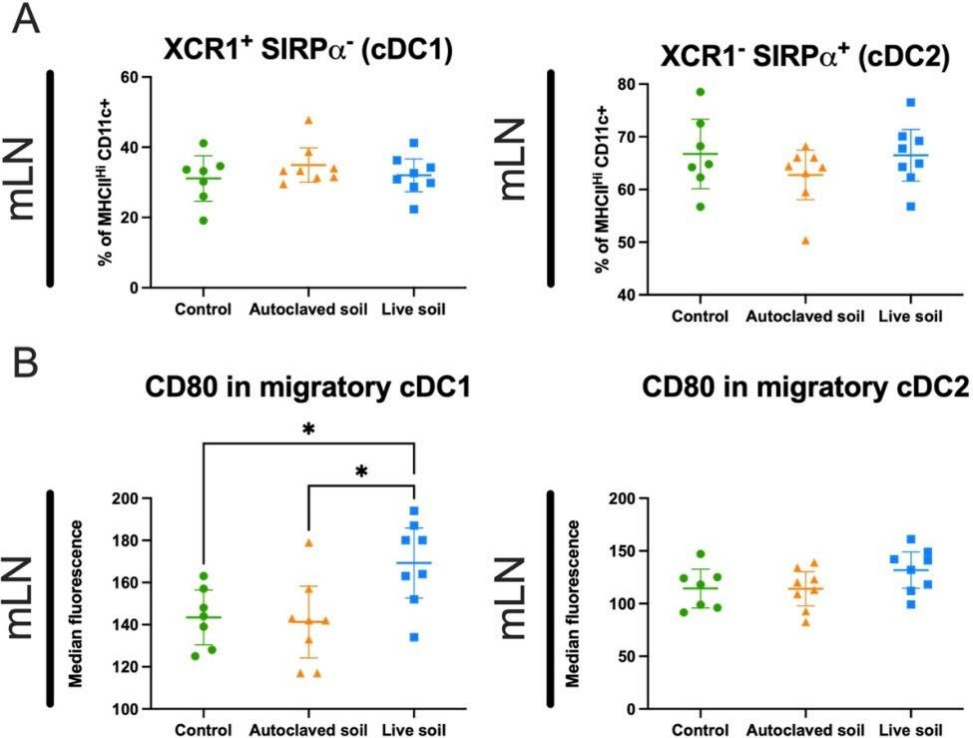
**Migratory conventional dendritic cells in mesenteric lymph nodes express an activated phenotype upon live soil exposure**. Spleen and mesenteric lymph nodes were collected at day 21 and myeloid cell compartments were analyzed using flow cytometry. Conventional dendritic cells 1 (cDC1) and 2 (cDC2) were defined as XCR1^+^SIRPα^−^ or XCR1^−^SIRPα^+^, respectively, and quantified from the from the CD11c^Int/Hi^MHCII^Hi^ population (**A**). The percentage of cDC1 and cDC2 showed no differences between mouse groups. (**B**) CD80 expression on cDC1 and cDC2 was quantified using median fluorescence intensity as a measure for activation. Data are shown as scatter plots with mean and 95% CI (n = 8 per group, from two independent exposure experiments). Each symbol represents an individual mouse, lines indicate the mean. The data was analyzed with One-way ANOVA with Tukey´s multiple comparison test, *p < 0.05

To characterize the immune status and identify possible immunomodulation induced by the treatments, we next characterized the myeloid compartment in gut-draining mLNs and spleen by flow cytometry. The analyzed cell types included dendritic cells, macrophages, monocytes, granulocytes and their subsets. Detailed different cell types, subsets and defining markers are presented in Supplementary Table 1. Gating strategy is shown in Supplementary Fig. 2. In mLN, we found that while there were no differences in the proportion of cDC1 and cDC2 (type 1 and type 2 conventional dendritic cells, respectively) the co-stimulatory molecule and activation marker CD80 was elevated in migratory cDC1 in the group that was exposed to live soil, when compared to control or autoclaved soil groups (p = 0.359 and p = 0.0177, respectively) (Fig. 2A-B). Migratory cDC1 dendritic cells are defined as CD11c^Int^MHCII^High^XCR1^+^SIRPα^−^, whereas migratory cDC2 cells are CD11c^High^MHCII^High^XCR1^−^SIRPα^+^. In spleen, no significant differences were found and overall, differences in cell subsets between treatments were minor. Third parameter Uniform manifold Approximation and projection for Dimension reduction (UMAP) plots are shown in Supplementary Fig. 1 for the myeloid cells (represented by mLN cells), demonstrating nearly identical subset composition.

### Lymphoid immune profile in mLN and spleen

**Fig. 3:**
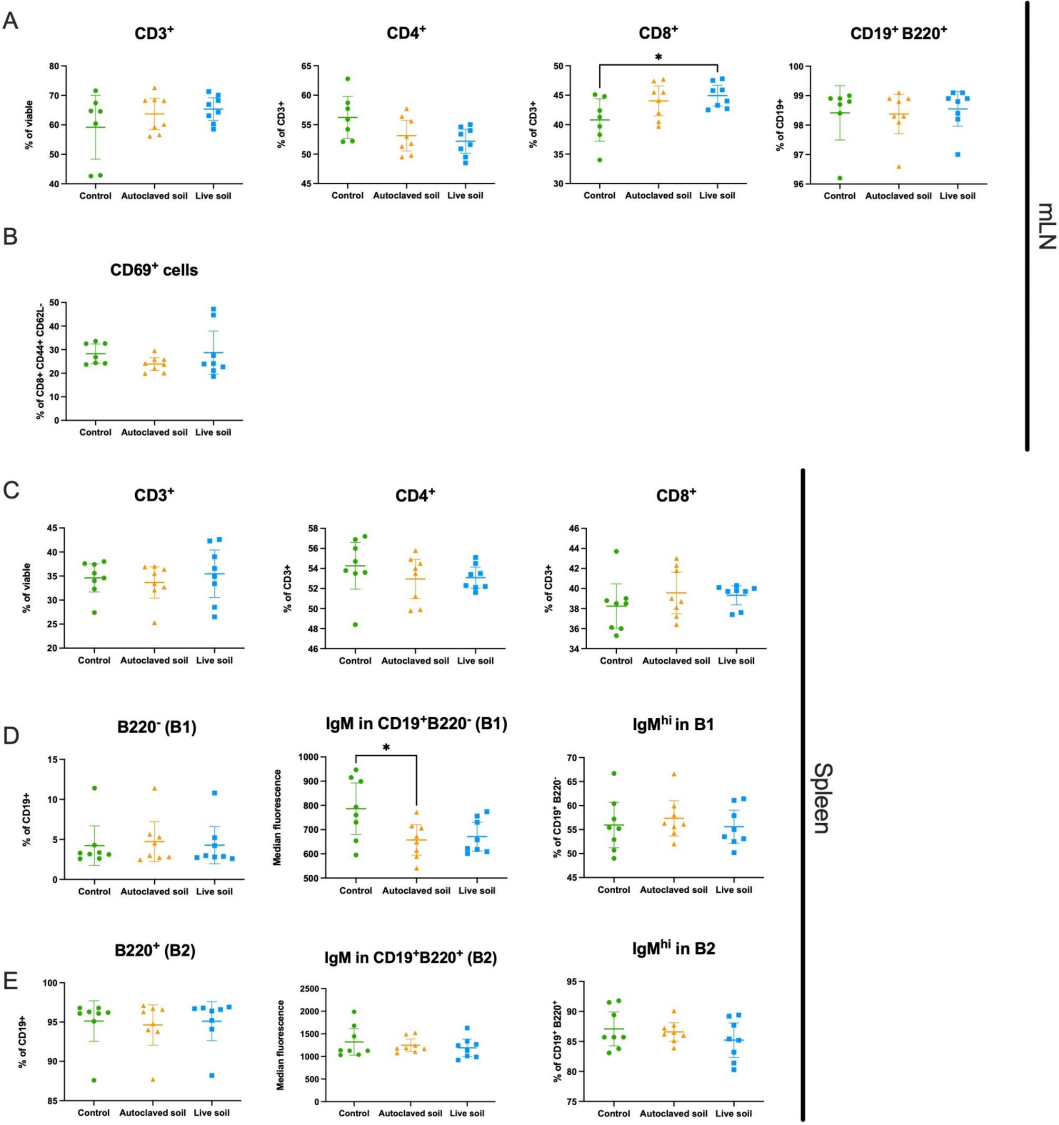
**Exposure to autoclaved soil downregulates IgM in B1 cells from spleen while live soil upregulates CD8**^**+**^**T cells in mesenteric lymph nodes**. Spleen and mesenteric lymph nodes were collected at day 21 and lymphoid cell compartments were analyzed using flow cytometry. **(A)** T cells (CD3^+^), helper T cells (CD3^+^ CD4^+^), cytotoxic T cells (CD3^+^ CD8^+^) and B cells (CD19^+^) in the mLN. **(B)** The percentage of CD69 + effector cytotoxic T cells (CD8^+^ CD44^+^ CD62L^−^) in the mLN. **(C)** CD3^+^, CD4^+^, CD8^+^ and CD19^+^ cells in spleen. **(D)** B1 cells (CD19^+^ B220^−^) and IgM expression on B1 cells in spleen. MFI was measured from total B1 population. **(E)** B2 cells (CD19^+^ B220^+^) and IgM expression on B2 cells in spleen. MFI was measured from total B2 population. Data are shown as scatter plots with mean and 95% CI (n = 8 per group, from two independent exposure experiments). Each symbol represents an individual mouse, lines indicate the mean. The data was analyzed with One-way ANOVA with Tukey´s multiple comparison test, *p < 0.05

We next analyzed the effect of soil exposure to the lymphoid compartment by flow cytometry. Cells were stained for markers separating T cells and B cells and their subsets. Detailed subsets and markers are depicted in Supplementary Tables 2 and gating strategy is shown in Supplementary Fig. 3. In mLN, we saw an increase of cytotoxic CD8^+^ of total CD3^+^ cells, (Fig. 3A). The difference was seen when control group was compared to the live soil group (p = 0.0417). Additionally, the cytotoxic effector memory population (CD8^+^CD44^+^CD62L^−^) in autoclaved soil group seemed to have a trend of decreased expression of the early activation marker CD69, but this was not statistically significant (p = 0.46; Fig. 3B). In spleen there were no differences in the proportion of CD3^+^, CD3^+^CD4^+^, CD3^+^CD8^+^ nor B1 or B2 cells (defined as CD3^−^CD19^+^ B220^−^ and CD3^−^CD19^+^B220^+^, respectively) (Fig. 3C-E), but IgM expression in B1 cells decreased after exposure to autoclaved soil compared to the control group (p = 0.0319) (Fig. 3D). As with the myeloid compartment, lymphoid cell composition was rather similar in mLN and spleen regardless of the treatment, as exemplified by third parameter UMAPs of splenic cells in Supplementary Fig. 1.

### Splenocyte activation response

**Fig. 4:**
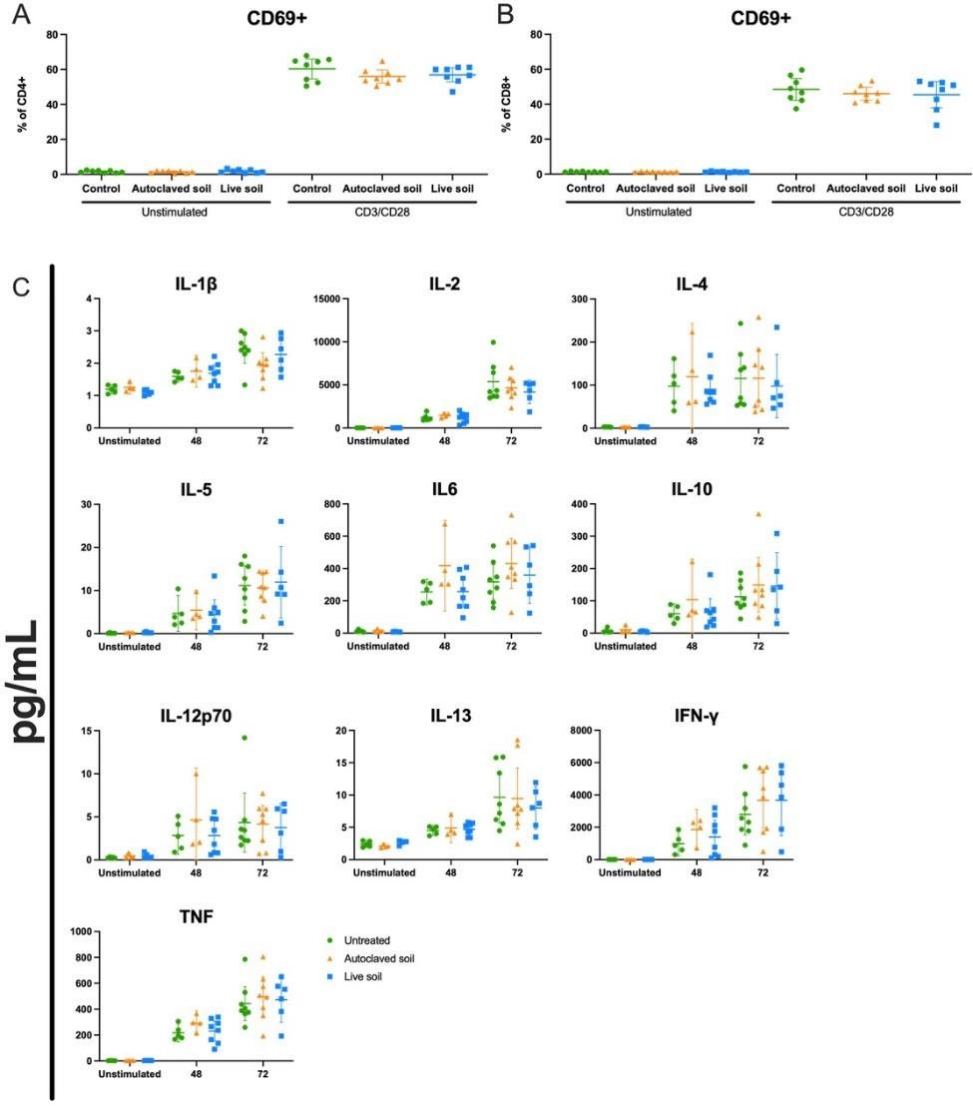
**T Cytokine secretion is not affected by exposure to autoclaved or live soil in an anti-CD3/anti-CD28 activation assay.** On day 21, single-cell suspension from splenocytes was prepared, cells were rested overnight and then either left unstimulated or stimulated with anti-CD3/anti-CD28 antibody cocktail. CD69 expression was assessed at 24 h post stimulation using flow cytometry for **(A)** CD4^+^ T cells and **(B)** CD8^+^ T cells. **(C)** Supernatants from unstimulated or stimulated splenocytes were collected at 48 and 72 h and analyzed using U-Plex from Meso Scale Discovery. Each symbol represents an individual mouse, lines indicate the mean 95% C.I. (n = 4 for Unstimulated and 48 h treatment, and n = 8 for 72 h, from two independent exposure experiments). The data was analyzed with One-way ANOVA

We previously observed that splenocytes from animals exposed to autoclaved soil powder expressed less pro-inflammatory cytokines when stimulated by PMA and ionomycin in vitro [39]. We now stimulated splenocytes from the different treatment groups with a more physiologically relevant anti-CD3/anti-CD28 antibody activation. T cell response was measured by flow cytometry using CD69, CD25, PD-1 and CTLA-4 expression. We found no significant differences in the percentage of activation nor in the magnitude of these markers mean fluorescence intensity at 24 h post-stimulation (Fig. 4A-B, CD69 only shown) when compared to control group. Moreover, we analyzed the profile of cytokine secretion (IL-1β, IL-2, IL-4, IL-5, IL-6, IL-10, IL-12p70, IL-13, IFN-γ, TNF) on the supernatants at different time points (Fig. 4C). No significant differences were found in the different cytokines at 48- or 72-hours post-stimulation. IL-17 F, IL-21, IL-25, IL-31 and IL-33 were not detected.

### Gene expression analysis of lung, small intestine, colon and mLN

**Fig. 5:**
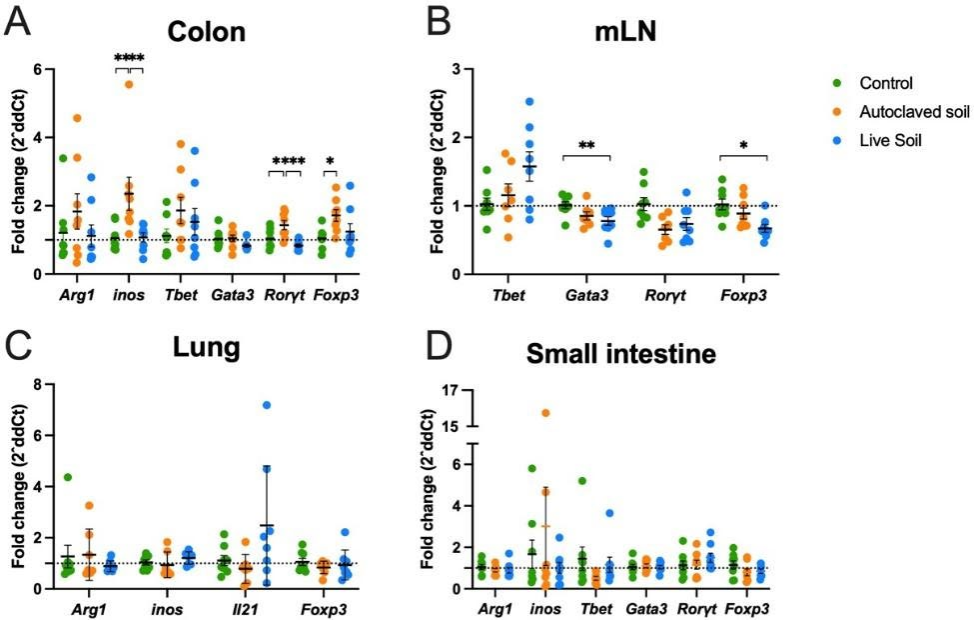
**In colon, exposure to autoclaved soil upregulates*inos, Rorγt*and*Foxp3*expression while live soil exposure downregulates*Rorγt***. **In mesenteric lymph nodes, live soil exposure downregulates*Gata3*and*Foxp3*expression.** Gene expression changes in different organs after no treatment, autoclaved or live soil exposure. RNA was extracted from the colon, mLN, lung or small intestine. Gene expression was measured using qPCR. (**A**) Macrophage activation associated genes (*Arg1* and *inos*) and T cell lineage associated genes (*Tbet, Gata3, Rorγt*, and *Foxp3*), in the colon. (**B**) T cell lineage-associated genes (*Tbet, Gata3, Rorγt*, and *Foxp3*) were measured in the mLN. (**C**) Macrophage activation associated genes (*Arg1* and *inos*), *Il21* cytokine and *Foxp3* expression were measured in the lung. (**D**) Macrophage activation associated genes (*Arg1* and *inos*) and T cell lineage associated genes (*Tbet, Gata3, Rorγt*, and *Foxp3*), in the small intestine. Values are shown as scatter plots with bar as mean and SEM (n = 8 per group, from two independent exposure experiments). Fold changes were calculated using control group mean and all data points were compared accordingly, including the control group. The data were analyzed with One-way ANOVA test with Tukey´s multiple comparison test, *p < 0.05, **p < 0.01

We then analyzed a set of genes important for the immune response in organs that could come in direct or indirect contact with the soil material or antigens from it, namely the colon, mesenteric lymph nodes, lung, and small intestine (Fig. 5). In the colon, *Inos* was significantly upregulated in autoclaved soil treated animals compared to the control and the live soil group (p = 0.0044 and 0.0046, respectively, Fig. 5A). Quite interestingly, *Rorγt* was upregulated in the autoclaved soil group when compared to the control and live soil group (p = 0.0347 and 0.0009, respectively). Autoclaved soil treated animals also showed higher levels of *Foxp3* expression compared to the control group (p = 0.0333), but it was not differently expressed in the live soil treated animals (Fig. 5A). In contrast to colon results, in the mLN, *Foxp3* and *Gata3* were significantly downregulated (p = 0.0039 and 0.0339, respectively) in the live soil treated animals compared to the control group, but no difference was found in the autoclaved soil-treated animals (Fig. 5B).

In the respiratory tract, no major differences were found in macrophage polarization genes *Arg1* and *Inos*, while the previously reported downregulation of *Il-21* was slightly reduced in autoclaved soil animals, but the difference was not statistically significant. (Fig. 5C). Finally, we measured the same macrophage associated genes in the small intestine without significant differences (Fig. 5D). T-cell polarization related transcription factors *Tbet*, *Gata3*, *Rorγt* and *Foxp3* did not indicate any significant changes (Fig. 5D).

## Discussion

The biodiversity hypothesis of immune-mediated diseases proposes that early life exposure to microbial diversity protects against immune-mediated diseases. To test this, immunomodulatory interventions to recapitulate natural microbial diversity in laboratory conditions without confounding factors are needed. In contrast to many previous studies and therapeutic interventions, which have focused on recapitulating existing microbiome and interventions using a single or a few microbial strains [27–32], our group has shown potentially beneficial immunological effects in human microbiota interventions using highly diverse intervention materials [34–38]. To dissect the molecular mechanisms behind these observed findings, animal models and the comprehensive immunological studies of different immunologically active organs and cells are needed. Even though Ottman et al. [40] did a single study using commercially manufactured soil enriched with a Bacillus strain, and we previously showed that autoclaved soil powder induced certain anti-inflammatory features in mice cultured in sterile conditions [39], the precise comparison of the effects of exposure to sterile versus live soil has been missing. In the current study, we compared the effects of autoclaved vs. non-autoclaved live soil powder on the immune system of WT B6 mice that were housed in non-sterile conditions. The current study can be considered as the first attempt to fill this crucial gap in knowledge.

Several details in our results support the hypothesis that both live and autoclaved soil trigger immune response. The notion that serum IFN-g level is elevated by live soil is logical. It suggests that microbial components in soil trigger intracellular type1 immune response. This is supported also by the fact that IL-12p70 levels are elevated in several mice from live soils or autoclaved soil groups, but in the control group none of the mice showed elevation of IL-12p70 expression (Fig. 1). On the other hand, the unchanged expression of most pro-inflammatory cytokines would support the non-toxic nature of both autoclaved and live soil preparates for mice. Interestingly, while alarmin IL-33 expression was not statistically different between the treatment groups, it is noteworthy that in all control group mice the expression of IL-33 was low, while there were several mice in autoclaved and live soil groups showing elevated expression of this alarmin. This further supports the hypothesis that both live and autoclaved soil has the potential to trigger immune response, in contrast to soil-free cages. Similar findings have been done using live or inactivated probiotics demonstrating that both formulas can be immunologically active and function as immunobiotics and postbiotics [41, 42].

Differences of the cellular landscape between control group and autoclaved or live soil exposed groups were subtle. Dendritic cells are critical sensors of antigen diversity by providing structural data of the antigens to CD4 T cells. In mice, myeloid DCs are classically divided to plasmacytoid DCs, and type 1 and type 2 DCs [43]. Of these, we found that activation marker CD80 was significantly elevated in migratory, non-autoclaved live soil-treated cDC1 cells. Functionally these cells regulate antigen cross-presentation, while cDC2s participate more in CD4 T cells differentiation [44]. This would be logical as these cells were likely activated by encountering the live soil-derived microbes. However, the fact that monocytes were not activated is somewhat confusing, suggesting that mammalian response to soil exposure is more complex than could be imagined based on the functions of individual antigens. In the lymphoid compartment, slight increase in the proportion of CD8^+^ T cells in the gut draining mLN from the live soil group was observed and could be related to the higher activation state of cDC1 cells. The notion that IgM levels were lower in B1 B cells from the spleen of autoclaved soil exposed mice is interesting. It might indicate B1 B cell activation and further differentiation to plasma cells. Natural IgM from B1 cells has been suggested to be important for homeostasis and immunity [45, 46], but also in certain cases promote inflammation by activating complement (discussed in [47]). Whether this might provide beneficial anti-inflammatory consequences, for example in experimental autoimmune models, remains to be studied.

Our results in colon and mLN indicate that exposure to live soil decreases *Gata3* and *Foxp3* expression in the mLN while autoclaved soil increased *Foxp3* expression in the colon. This is in contrast to small intestine, where no immunological effects were seen. *Gata3* and *Foxp3* downregulation in the mLN are in line with the type 1 response elicited by the live soil material [48], while the autoclaved, inactivated soil was not able to produce a similar effect. This is also supported by the elevated *Tbet* expression in several live soil treated animals. However, the increased *Foxp3* and *Rorγt* expression in the colon upon exposure to autoclaved material could indicate a local immunomodulatory and homeostatic effects in the absence of a stronger inflammatory reaction [49–51]. One possibility is that the antigen diversity provided in the soil also modulates the complex lymphocyte trafficking between tissues and secondary lymphoid organs in the gut [52]. The difference between small intestine and colon could also reflect the possible lower microbial flexibility of the upper intestine. Obviously, the current study was not designed to survey these dilemmas, but based on our findings it seems crucial to consider appropriate sampling procedures in future mammalian, including human, trials focusing on gut function and microbiota. Our previous work indicated relatively subtle changes in the gut microbiota, when mice were exposed to autoclaved soil in sterile environment [39]. For this, in this work we focused to host response to the treatment, rather than the microbiome composition. Future experiments will be needed to carefully assess the microbiome upon soil challenge. It is also quite interesting that a single bacterial species such as *Akkermansia muciniphila* in the gut may provide a protective tolerance against autoimmune reactions in mice [53].

One limitation of this study is the fact that we were unable to perform these experiments in similar sterile conditions as our previous work [39] due to risk of contaminating the Institutional Animal Facility using the non-autoclaved live material. For this, the exposure of the animals was done in less sterile conditions which may explain part of the differences between the current and our previous study. The exposure of the animals to environmental microbes in less sterile conditions might reduce differences between groups, as the animals would have a more mature and primed T cell phenotype at the onset of the experiment, instead of the naïve phenotype typical for animals housed in laboratory conditions [54]. Additionally, since the decrease in lung IL-21 mRNA expression with autoclaved soil was not statistically significant in mice housed in less sterile conditions (Fig. 5C), while the difference was found between exposed and control mice in sterile conditions in our earlier study [39], we speculate that airborne spores may play a role in the immune response of our mice model. Another limitation is that only female mice were used as we wanted to exclude the effect of gender in the analysis due to relatively small group sizes (n = 8).

Performing the experiment in less sterile environment reflects a more realistic situation. While still limited to an inbred mouse line, the less sterile environment allows us to realistically evaluate the efficacy of microbially rich and autoclaved soil on immunomodulation. It is also possible that the relatively small size of individual groups may have influenced the statistical power to detect modest changes between the groups. Future experiments elucidating the capability of this immunomodulation to counterbalance, for example, experimental allergic inflammation in mice, are well-warranted.

## Conclusions

In conclusion, live soil treatment seems to have immunological effects. It induced IFN-γ in serum, cDC1 activation and reduced *Gata3* expression in mLN. Some of the immunological outcomes differed between the tissues studied and the treatments the mice were exposed to. For example, *Foxp3* expression was elevated in the colons of mice exposed to autoclaved soil while it was reduced in mLNs from live soil-exposed animals. The reasons for these differences are not known, but they may reflect the distinct immunological functionalities of different sites, or alternatively, changes in microbial antigen content during autoclavation. The current study opens possibilities to tailor the effects of biodiversity-based immunomodulation by modulating the content and quality of the intervention material.

## Methods

*Animals and exposure to plant and soil-based extract*. The plant and soil-based powder used for the exposure was manufactured by the Laboratory of Environmental Ecology, University of Helsinki, as described previously [11, 34, 55]. It comprised of a mixture of sieved composted materials including six commercial gardening soils (trade names: Musta Multa, Niittymulta, Nurmikkomulta, Perennamulta, Puistomulta and Viljelymulta), deciduous leaf litter, peat and agricultural sludge, and dried and crushed Sphagnum moss.

A total of 24 female C57BL/6JRj mice from Janvier Labs (Le Genest-Saint-Isle, France) were used in this study. The mice were 7–8 weeks of age at the beginning of the exposure period. Animals were housed in groups of 4 animals in open ventilated cages with aspen bedding and cardboard cage enrichment and nests (Scanbur, Karlslunde, Denmark) at Natural Resources Institute Finland (Luke), Jokioinen. The animals were exposed daily to soil material (inactivated by autoclaving or live, non-autoclaved soil) for one hour, five days a week for three weeks (21 days). For the exposure, the mice were transferred to a new clean cage (floor 360 cm2) with 300 mL of clean aspen bedding and 50 mL of lyophilized soil material sprinkled on top. Control animals were placed in a new cage with clean bedding only, for one hour. The lyophilized soil samples were dusty and visibly adhered to mice during the exposure. The animals from different groups (autoclaved soil, live soil or control) were fed with the same food (SAFE D113, Safe, Rosenberg, Germany) and autoclaved drinking water *ad libitum*, but were kept in different rooms and the researchers handling the mice changed single use protection clothes every time before handling animals of different groups to avoid cross contamination. During the exposure, the cage with mice was kept in a hood to avoid contamination by soil dust and afterwards the hood was cleaned with 70% ethanol. At day 21 mice were euthanized in a CO_2_ chamber and organs were subsequently collected for analysis. The experiment was performed twice, with 4 animals per group (autoclaved soil, live soil or control) both times.

*Flow cytometry*. Single cell suspensions of spleen and mLN were prepared by mechanic dissociation of the organs with a 10 mL syringe plunger and 40 μm cell strainer (Thermo Fisher Scientific, Waltham, MA, US) in PBS^−/−^ buffer (pH 7.2, Thermo Fisher Scientific) supplemented with 2% FBS and 2 mM EDTA. The red blood cells of the spleen samples were lysed with 1 min ACK (Lonza, Basel, Switzerland) treatment. Single cell suspensions were treated with Rat Anti-Mouse CD16/CD32 antibodies (Mouse BD Fc Block™, Becton Dickinson Biosciences, Franklin Lakes, NJ) for 5 min at 4 °C before staining. The cells from spleens and lymph nodes were divided into two panels with an emphasis on either myeloid cells (B220, CD3, CD11b, CD11c, CD64, CD80, CD103, CD169, F4/80, ICAM-1(CD54), Ly6C, MHCII, SIRPα (CD172), XCR1, modified from [56]) or lymphoid cells (B220, CD3, CD4, CD8, CD19, CD25, CD62L, CD44, CD69, CD127, IgM, Ly6G). Some splenocytes were activated (see below) before staining for B220, CD3, CD4, CD8, CD25, CD49b, CD69, CTLA-4, MHCII and PD-1 (CD279). Dead cells were stained with Fixable Viability Stain 780 (BD) for 15 min in PBS, RT. Antibody clone and manufacturer details are listed in Supplementary Table 3. After washing with 2% FBS, 1 mM EDTA, 0,05% NaN_3_ in PBS, cells were incubated with the antibody cocktail in BD Horizon Buffer for 20 min at 4 °C and then washed twice. Samples of the myeloid and lymphoid panel were fixed with 2% paraformaldehyde in PBS for 10 min, RT, washed twice and kept in washing buffer until analysis. Activated splenocytes were analyzed freshly after staining. All samples were run with BD FACSAria Fusion and subsequent data analysis was performed with FlowJo software (BD).

*RNA extraction and RT-qPCR analysis*. Spleen, mesenteric lymph node, lung, small intestine and colon samples were collected at day 21 and stored in RNALater (Thermo Fisher Scientific) after collection and stored at -80 °C until further use. Before RNA extraction, organs were homogenized using ceramic beads (VWR. Precellys®, CKMIX) with PowerLyzer® 24 homogenizer (Bertin Instruments, Bertin Technologies SAS, France) at 3200 rpm, 45 s, 3 times. The RNA was extracted using E.Z.N.A.® Total RNA Kit I (Omega Bio-tek, Inc.). Synthesis of cDNA from RNA samples was performed using iScript™ cDNA Synthesis Kit (Biorad, Hercules, CA, US). Gene expression was assessed using RT-qPCR. The qPCR reaction was prepared using SYBR Green Luna® Universal qPCR Master Mix (New England Biolabs Inc. Ipswich, MA, US) and samples were analyzed with ABI ABI QuantStudio 12 K Flex System (Thermo Fisher Scientific). In brief, three technical replicates were performed for each biological sample. Then, delta Ct values (dCt) were calculated using the difference between the target genes Ct averages to the 18s-rRNA Ct average value for each sample respectively. Later, ddCt values were obtained subtracting the dCt value for each sample to the dCt average value of the control group (including each sample from the control group compared to the control group mean). Finally, fold changes were calculated using the following formula: 2^-ddCt.

*T cell activation assay*. Spleens from animals were collected after euthanasia and single cell suspension was obtained as described above, after which the cells were rested overnight in RPMI 1640 with 10% FBS (Thermo Fisher Scientific), 1% L-glutamine (Lonza), and 1% penicillin/streptomycin (Lonza). The next day, splenocytes were stimulated on plates pre-coated with anti-CD3 (0,5 ug/mL) and anti-CD28 (1 ug/mL). Control cells were left untreated. Supernatants were collected at 48- and 72-hours post-stimulation and stored at -80 °C for cytokine measurement. A part of the cells was harvested for flow cytometry at 24 h time point.

*Serum and supernatant cytokine analysis*. Mouse sera were collected by cardiac puncture after euthanasia and stored at -80 °C until further use. Supernatants were collected from activated splenocytes as described above. Cytokine quantification for IFN-γ, IL-1β, IL-2, IL-4, IL-5, IL-6, IL-10, IL-12p70, IL-13, IL-17E/IL-25, IL17F, IL-21, IL-31, IL-33 and TNF was performed using a custom version of U-PLEX Biomarker Group 1 (ms) Assay and analyzed with MESO Quickplex SQ 120 according to manufacturer’s instructions (kit and instrument by Meso Scale Discovery, Rockville, MD, US).

*Statistical analysis*. Normal distribution was assessed for each individual data set using Anderson-Darling test (alpha = 0.05). Non-parametric data were analyzed using a Kruskal-Wallis (alpha = 0.05, two tailed), parametric data were analyzed using a One-way ANOVA, as appropriate (alpha = 0.05, two tailed). All immunological data sets were analyzed using GraphPad Prism 8.0 (GraphPad Inc., USA).

### Electronic supplementary material

Below is the link to the electronic supplementary material.

Supplementary Material 1

## Data Availability

All data and materials are available for qualified researchers upon request. Contact person for access to the raw data: Ilkka Junttila, ilkka.junttila@tuni.fi.

## Abbreviations

IL: Interleukin
LN: lymph node
mLN: mesenteric lymph node
Treg: regulatory T cell
TGF-b: Transforming growth factor beta
CD: Cluster of differentiation
DC: dendritic cell
GM-CSF: Granulocyte-macrophage colony-stimulating factor
TNF: Tumor necrosis factor
UMAP: Uniform manifold Approximation and projection for Dimension reduction

## References

[1] Cryan JF, O’Riordan KJ, Sandhu K, Peterson V, Dinan TG (2020). The gut microbiome in neurological disorders. Lancet Neurol.

[2] Dinan TG, Cryan JF (2017). The Microbiome-Gut-Brain Axis in Health and Disease. Gastroenterol Clin North Am.

[3] Haahtela T (2019). A biodiversity hypothesis. Allergy.

[4] Haahtela T, Alenius H, Lehtimäki J, Sinkkonen A, Fyhrquist N, Hyöty H (2021). Immunological resilience and biodiversity for prevention of allergic diseases and asthma. Allergy.

[5] Hanski I, von Hertzen L, Fyhrquist N, Koskinen K, Torppa K, Laatikainen T (2012). Environmental biodiversity, human microbiota, and allergy are interrelated. Proc Natl Acad Sci U S A.

[6] Kondrashova A, Seiskari T, Ilonen J, Knip M, Hyöty H (2013). The Hygiene hypothesis and the sharp gradient in the incidence of autoimmune and allergic diseases between russian Karelia and Finland. APMIS.

[7] Noce A, Marrone G, Di Daniele F, Ottaviani E, Wilson Jones G, Bernini R (2019). Impact of Gut Microbiota Composition on Onset and Progression of Chronic Non-Communicable Diseases. Nutrients.

[8] Rooney CM, Mankia K, Emery P (2020). The role of the Microbiome in driving RA-Related autoimmunity. Front Cell Dev Biol.

[9] Hui N, Parajuli A, Puhakka R, Grönroos M, Roslund MI, Vari HK (2019). Temporal variation in indoor transfer of dirt-associated environmental bacteria in agricultural and urban areas. Environ Int.

[10] Parajuli A, Grönroos M, Siter N, Puhakka R, Vari HK, Roslund MI (2018). Urbanization reduces transfer of Diverse Environmental Microbiota Indoors. Front Microbiol.

[11] Hui N, Grönroos M, Roslund MI, Parajuli A, Vari HK, Soininen L (2019). Diverse Environmental Microbiota as a Tool to augment Biodiversity in Urban Landscaping materials. Front Microbiol.

[12] Lehtimäki J, Karkman A, Laatikainen T, Paalanen L, von Hertzen L, Haahtela T (2017). Patterns in the skin microbiota differ in children and teenagers between rural and urban environments. Sci Rep.

[13] Lozupone CA, Stombaugh JI, Gordon JI, Jansson JK, Knight R (2012). Diversity, stability and resilience of the human gut microbiota. Nature.

[14] Parajuli A, Hui N, Puhakka R, Oikarinen S, Grönroos M, Selonen VAO (2020). Yard vegetation is associated with gut microbiota composition. Sci Total Environ.

[15] Rook GA (2013). Regulation of the immune system by biodiversity from the natural environment: an ecosystem service essential to health. Proc Natl Acad Sci U S A.

[16] Brugman S, Perdijk O, van Neerven RJJ, Savelkoul HFJ (2015). Mucosal Immune Development in Early Life: setting the stage. Arch Immunol Ther Exp (Warsz).

[17] Kim H, Sitarik AR, Woodcroft K, Johnson CC, Zoratti E (2019). Birth Mode, Breastfeeding, Pet exposure, and antibiotic use: Associations with the gut microbiome and sensitization in children. Curr Allergy Asthma Rep.

[18] Koskela HO, Happonen KK, Remes ST, Pekkanen J (2005). Effect of farming environment on sensitisation to allergens continues after childhood. Occup Environ Med.

[19] Lehtimäki J, Thorsen J, Rasmussen MA, Hjelmsø M, Shah S, Mortensen MS (2021). Urbanized microbiota in infants, immune constitution, and later risk of atopic diseases. J Allergy Clin Immunol.

[20] Nurminen N, Cerrone D, Lehtonen J, Parajuli A, Roslund M, Lönnrot M (2021). Land Cover of Early-Life Environment modulates the risk of type 1 diabetes. Diabetes Care.

[21] Okada H, Kuhn C, Feillet H, Bach JF (2010). The hygiene hypothesis for autoimmune and allergic diseases: an update. Clin Exp Immunol.

[22] Ruokolainen L, von Hertzen L, Fyhrquist N, Laatikainen T, Lehtomäki J, Auvinen P (2015). Green areas around homes reduce atopic sensitization in children. Allergy.

[23] Flandroy L, Poutahidis T, Berg G, Clarke G, Dao MC, Decaestecker E (2018). The impact of human activities and lifestyles on the interlinked microbiota and health of humans and of ecosystems. Sci Total Environ.

[24] Kim JE, Kim HS (2019). Microbiome of the skin and gut in atopic dermatitis (AD): understanding the pathophysiology and finding Novel Management Strategies. J Clin Med.

[25] Ott SJ, Musfeldt M, Wenderoth DF, Hampe J, Brant O, Fölsch UR (2004). Reduction in diversity of the colonic mucosa associated bacterial microflora in patients with active inflammatory bowel disease. Gut.

[26] Cohen NA, Maharshak N (2017). Novel indications for fecal microbial transplantation: update and review of the literature. Dig Dis Sci.

[27] Abrahamsson TR, Jakobsson T, Björkstén B, Oldaeus G, Jenmalm MC (2013). No effect of probiotics on respiratory allergies: a seven-year follow-up of a randomized controlled trial in infancy. Pediatr Allergy Immunol.

[28] Gallo A, Passaro G, Gasbarrini A, Landolfi R, Montalto M (2016). Modulation of microbiota as treatment for intestinal inflammatory disorders: an uptodate. World J Gastroenterol.

[29] McFarland LV (2014). Use of probiotics to correct dysbiosis of normal microbiota following disease or disruptive events: a systematic review. BMJ Open.

[30] Mennini M, Dahdah L, Artesani MC, Fiocchi A, Martelli A (2017). Probiotics in Asthma and Allergy Prevention. Front Pediatr.

[31] Prakoeswa CRS, Herwanto N, Prameswari R, Astari L, Sawitri S, Hidayati AN (2017). Lactobacillus plantarum IS-10506 supplementation reduced SCORAD in children with atopic dermatitis. Benef Microbes.

[32] Stiemsma LT, Reynolds LA, Turvey SE, Finlay BB (2015). The hygiene hypothesis: current perspectives and future therapies. Immunotargets Ther.

[33] Tischer C, Kirjavainen P, Matterne U, Tempes J, Willeke K, Keil T (2022). Interplay between natural environment, human microbiota and immune system: a scoping review of interventions and future perspectives towards allergy prevention. Sci Total Environ.

[34] Nurminen N, Lin J, Grönroos M, Puhakka R, Kramna L, Vari HK (2018). Nature-derived microbiota exposure as a novel immunomodulatory approach. Future Microbiol.

[35] Roslund MI, Puhakka R, Grönroos M, Nurminen N, Oikarinen S, Gazali AM (2020). Biodiversity intervention enhances immune regulation and health-associated commensal microbiota among daycare children. Sci Adv.

[36] Roslund MI, Parajuli A, Hui N, Puhakka R, Grönroos M, Soininen L (2022). A placebo-controlled double-blinded test of the biodiversity hypothesis of immune-mediated diseases: environmental microbial diversity elicits changes in cytokines and increase in T regulatory cells in young children. Ecotoxicol Environ Saf.

[37] Soininen L, Roslund MI, Nurminen N, Puhakka R, Laitinen OH, Hyöty H (2022). Indoor green wall affects health-associated commensal skin microbiota and enhances immune regulation: a randomized trial among urban office workers. Sci Rep.

[38] Roslund MI, Puhakka R, Nurminen N, Oikarinen S, Siter N, Grönroos M (2021). Long-term biodiversity intervention shapes health-associated commensal microbiota among urban day-care children. Environ Int.

[39] González-Rodríguez MI, Nurminen N, Kummola L, Laitinen OH, Oikarinen S, Parajuli A (2022). Effect of inactivated nature-derived microbial composition on mouse immune system. Immun Inflamm Dis.

[40] Ottman N, Ruokolainen L, Suomalainen A, Sinkko H, Karisola P, Lehtimäki J (2019). Soil exposure modifies the gut microbiota and supports immune tolerance in a mouse model. J Allergy Clin Immunol.

[41] Sanaei M, Mahdavi M, Setayesh N, Shahverdi AR, Sepehrizadeh Z, Yazdi MH (2021). Comparison of Cytokine expression in human PBMCs stimulated with normal and heat-shocked Lactobacillus plantarum cell lysate. Probiotics Antimicrob Proteins.

[42] Zago M, Massimiliano L, Bonvini B, Penna G, Giraffa G, Rescigno M (2021). Functional characterization and immunomodulatory properties of Lactobacillus helveticus strains isolated from italian hard cheeses. PLoS ONE.

[43] Cabeza-Cabrerizo M, Cardoso A, Minutti CM, Pereira da Costa M (2021). Reis e Sousa C. Dendritic cells revisited. Annu Rev Immunol.

[44] Perner C, Flayer CH, Zhu X, Aderhold PA, Dewan ZNA, Voisin T (2020). Substance P release by sensory neurons Triggers Dendritic Cell Migration and initiates the Type-2 Immune response to Allergens. Immunity.

[45] Yang Y, Tung JW, Ghosn EEB, Herzenberg LA, Herzenberg LA (2007). Division and differentiation of natural antibody-producing cells in mouse spleen. Proc Natl Acad Sci USA.

[46] Choi YS, Baumgarth N (2008). Dual role for B-1a cells in immunity to influenza virus infection. J Exp Med.

[47] Prieto JMB, Felippe MJB (2017). Development, phenotype, and function of non-conventional B cells. Comp Immunol Microbiol Infect Dis.

[48] Colamatteo A, Carbone F, Bruzzaniti S, Galgani M, Fusco C, Maniscalco GT (2020). Molecular Mechanisms Controlling Foxp3 expression in Health and Autoimmunity: from epigenetic to post-translational regulation. Front Immunol.

[49] Song X, Sun X, Oh SF, Wu M, Zhang Y, Zheng W (2020). Microbial bile acid metabolites modulate gut RORγ + regulatory T cell homeostasis. Nature.

[50] Yang BH, Hagemann S, Mamareli P, Lauer U, Hoffmann U, Beckstette M (2016). Foxp3 + T cells expressing RORγt represent a stable regulatory T-cell effector lineage with enhanced suppressive capacity during intestinal inflammation. Mucosal Immunol.

[51] Ohnmacht C (2016). Tolerance to the intestinal microbiota mediated by ROR(γt) + cells. Trends Immunol.

[52] Habtezion A, Nguyen LP, Hadeiba H, Butcher EC (2016). Leukocyte trafficking to the small intestine and Colon. Gastroenterology.

[53] Hänninen A, Toivonen R, Pöysti S, Belzer C, Plovier H, Ouwerkerk JP (2018). *Akkermansia muciniphila* induces gut microbiota remodelling and controls islet autoimmunity in NOD mice. Gut.

[54] Beura LK, Hamilton SE, Bi K, Schenkel JM, Odumade OA, Casey KA (2016). Normalizing the environment recapitulates adult human immune traits in laboratory mice. Nature.

[55] Grönroos M, Parajuli A, Laitinen OH, Roslund MI, Vari HK, Hyöty H (2019). Short-term direct contact with soil and plant materials leads to an immediate increase in diversity of skin microbiota. Microbiologyopen.

[56] DiPiazza AT, Hill JP, Graham BS, Ruckwardt TJ (2019). OMIP-061: 20-Color Flow Cytometry Panel for High-Dimensional characterization of Murine Antigen-Presenting cells. Cytometry A.

