# Supplementary material for: Comparison of the effect of autoclaved and non-autoclaved live soil exposure on the mouse immune system: Effect of soil exposure on immune system
Source: BMC Immunol. 2023 Sep 9;24:29. doi: 10.1186/s12865-023-00565-0 (PMC10492337; doi:10.1186/s12865-023-00565-0)
Supplement: Supplementary file 1 — Supplementary Material 1 [file 12865_2023_565_MOESM1_ESM.pptx]

## Slide 1
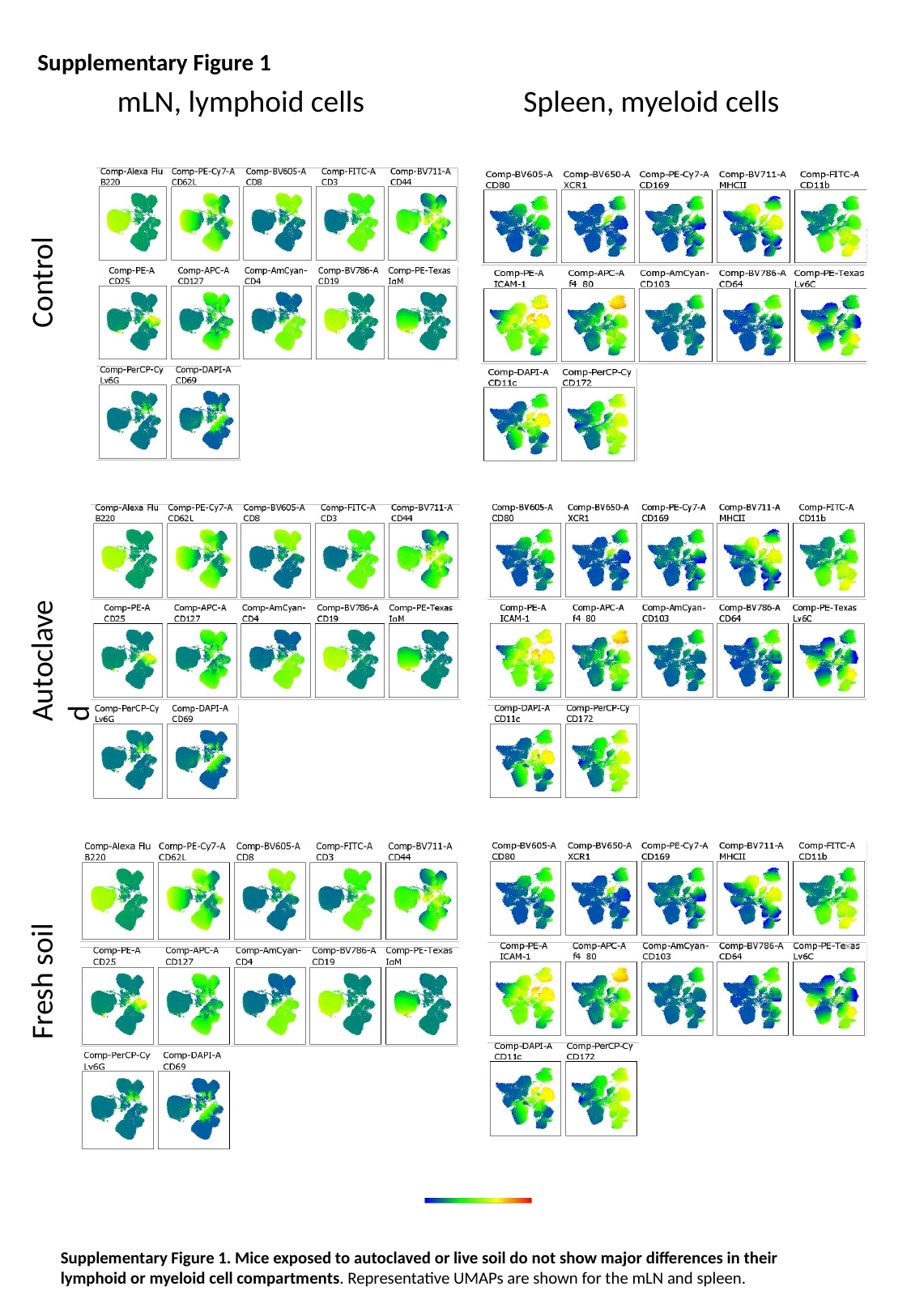

Supplementary Figure 1
mLN, lymphoid cells
Spleen, myeloid cells
Control
Autoclaved
Fresh soil
Supplementary Figure 1. Mice exposed to autoclaved or live soil do not show major differences in their lymphoid or myeloid cell compartments. Representative UMAPs are shown for the mLN and spleen.

## Slide 2
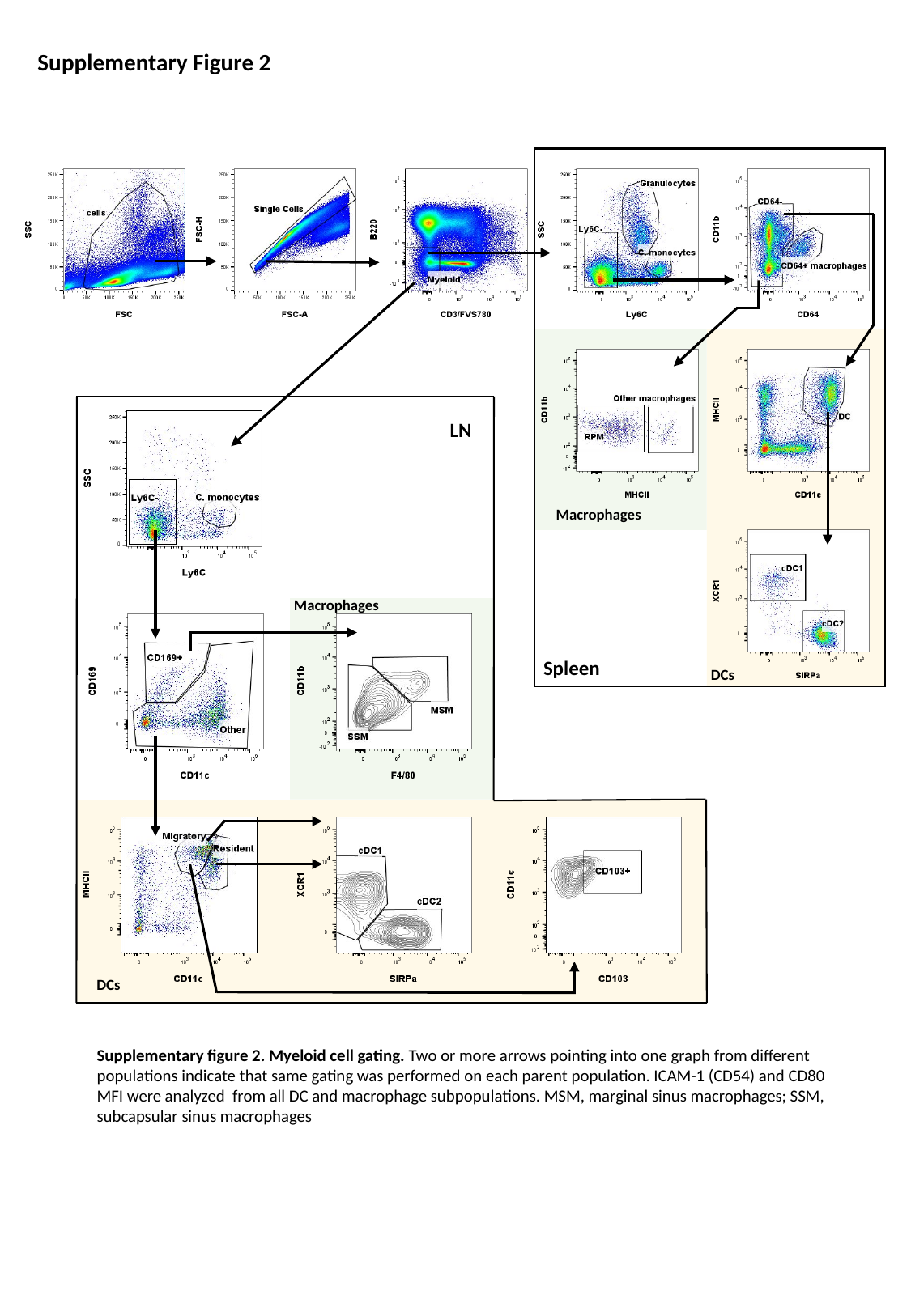

Supplementary Figure 2
LN
Macrophages
Macrophages
Spleen
DCs
DCs
Supplementary figure 2. Myeloid cell gating. Two or more arrows pointing into one graph from different populations indicate that same gating was performed on each parent population. ICAM-1 (CD54) and CD80 MFI were analyzed from all DC and macrophage subpopulations. MSM, marginal sinus macrophages; SSM, subcapsular sinus macrophages

## Slide 3
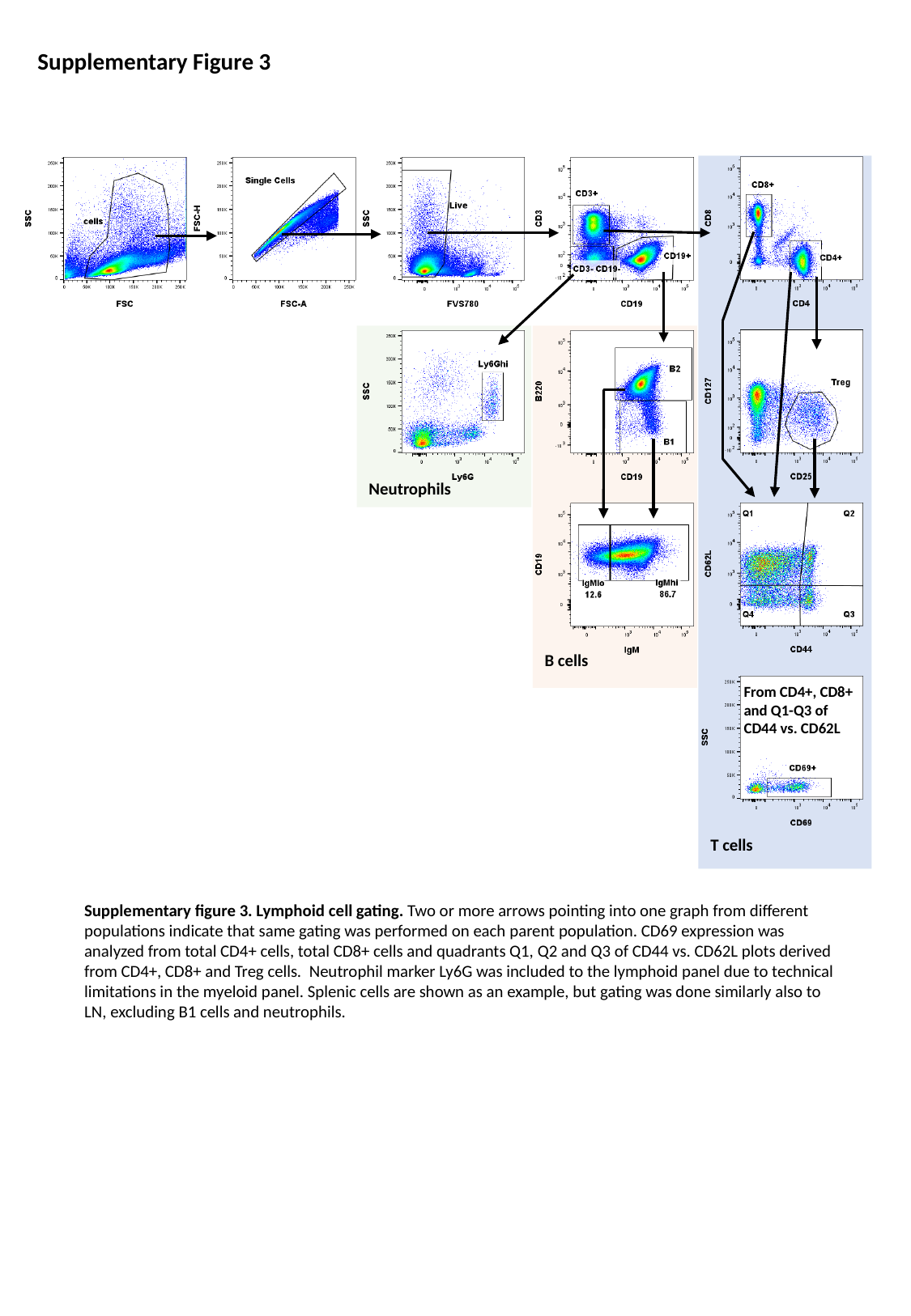

Supplementary Figure 3
Neutrophils
B cells
From CD4+, CD8+ and Q1-Q3 of CD44 vs. CD62L
T cells
Supplementary figure 3. Lymphoid cell gating. Two or more arrows pointing into one graph from different populations indicate that same gating was performed on each parent population. CD69 expression was analyzed from total CD4+ cells, total CD8+ cells and quadrants Q1, Q2 and Q3 of CD44 vs. CD62L plots derived from CD4+, CD8+ and Treg cells. Neutrophil marker Ly6G was included to the lymphoid panel due to technical limitations in the myeloid panel. Splenic cells are shown as an example, but gating was done similarly also to LN, excluding B1 cells and neutrophils.

## Slide 4
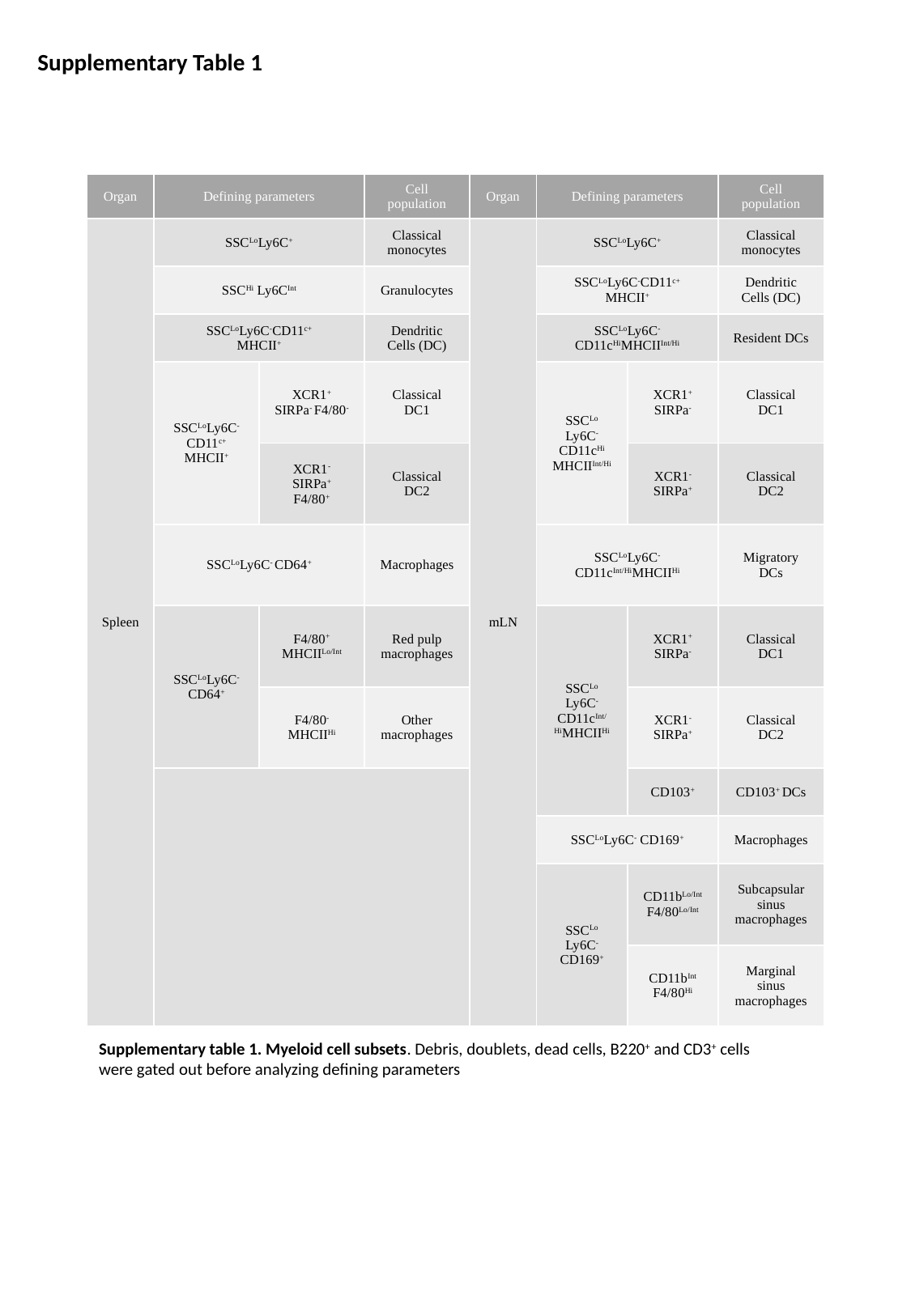

Supplementary Table 1
| Organ | Defining parameters | | Cell population | Organ | Defining parameters | | Cell population |
| --- | --- | --- | --- | --- | --- | --- | --- |
| Spleen | SSCLoLy6C+ | | Classical monocytes | mLN | SSCLoLy6C+ | | Classical monocytes |
| Spleen | SSCHi Ly6CInt | | Granulocytes | mLN | SSCLoLy6C-CD11c+ MHCII+ | | Dendritic Cells (DC) |
| | SSCLoLy6C-CD11c+ MHCII+ | | Dendritic Cells (DC) | | SSCLoLy6C- CD11cHiMHCIIInt/Hi | | Resident DCs |
| | SSCLoLy6C-CD11c+ MHCII+ | XCR1+ SIRPa- F4/80- | Classical DC1 | | SSCLo Ly6C- CD11cHi MHCIIInt/Hi | XCR1+ SIRPa- | Classical DC1 |
| | | XCR1- SIRPa+ F4/80+ | Classical DC2 | | | XCR1- SIRPa+ | Classical DC2 |
| | SSCLoLy6C- CD64+ | | Macrophages | | SSCLoLy6C- CD11cInt/HiMHCIIHi | | Migratory DCs |
| | SSCLoLy6C- CD64+ | F4/80+ MHCIILo/Int | Red pulp macrophages | | SSCLo Ly6C- CD11cInt/HiMHCIIHi | XCR1+ SIRPa- | Classical DC1 |
| | | F4/80- MHCIIHi | Other macrophages | | | XCR1- SIRPa+ | Classical DC2 |
| | | | | | | CD103+ | CD103+ DCs |
| | | | | | SSCLoLy6C- CD169+ | | Macrophages |
| | | | | | SSCLo Ly6C- CD169+ | CD11bLo/Int F4/80Lo/Int | Subcapsular sinus macrophages |
| | | | | | | CD11bInt F4/80Hi | Marginal sinus macrophages |
Supplementary table 1. Myeloid cell subsets. Debris, doublets, dead cells, B220+ and CD3+ cells were gated out before analyzing defining parameters

## Slide 5
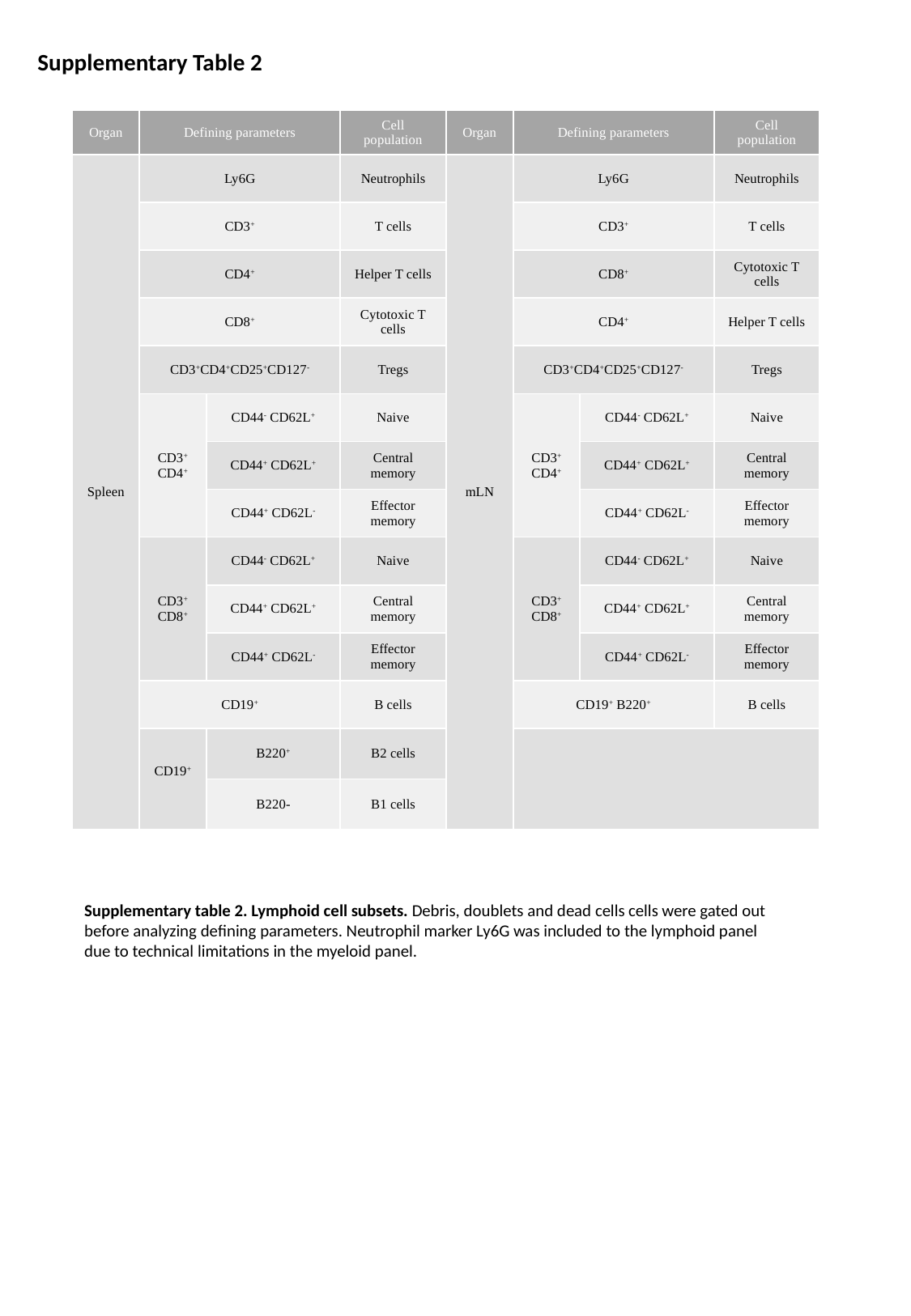

Supplementary Table 2
| Organ | Defining parameters | | Cell population | Organ | Defining parameters | | Cell population |
| --- | --- | --- | --- | --- | --- | --- | --- |
| Spleen | Ly6G | | Neutrophils | mLN | Ly6G | | Neutrophils |
| Spleen | CD3+ | | T cells | mLN | CD3+ | | T cells |
| Spleen | CD4+ | | Helper T cells | mLN | CD8+ | | Cytotoxic T cells |
| | CD8+ | | Cytotoxic T cells | | CD4+ | | Helper T cells |
| | CD3+CD4+CD25+CD127- | | Tregs | | CD3+CD4+CD25+CD127- | | Tregs |
| | CD3+ CD4+ | CD44- CD62L+ | Naive | | CD3+ CD4+ | CD44- CD62L+ | Naive |
| | | CD44+ CD62L+ | Central memory | | | CD44+ CD62L+ | Central memory |
| | | CD44+ CD62L- | Effector memory | | | CD44+ CD62L- | Effector memory |
| | CD3+ CD8+ | CD44- CD62L+ | Naive | | CD3+ CD8+ | CD44- CD62L+ | Naive |
| | | CD44+ CD62L+ | Central memory | | | CD44+ CD62L+ | Central memory |
| | | CD44+ CD62L- | Effector memory | | | CD44+ CD62L- | Effector memory |
| | CD19+ | | B cells | | CD19+ B220+ | | B cells |
| | CD19+ | B220+ | B2 cells | | | | Transitional B cells |
| | | B220- | B1 cells | | | | |
Supplementary table 2. Lymphoid cell subsets. Debris, doublets and dead cells cells were gated out before analyzing defining parameters. Neutrophil marker Ly6G was included to the lymphoid panel due to technical limitations in the myeloid panel.

## Slide 6
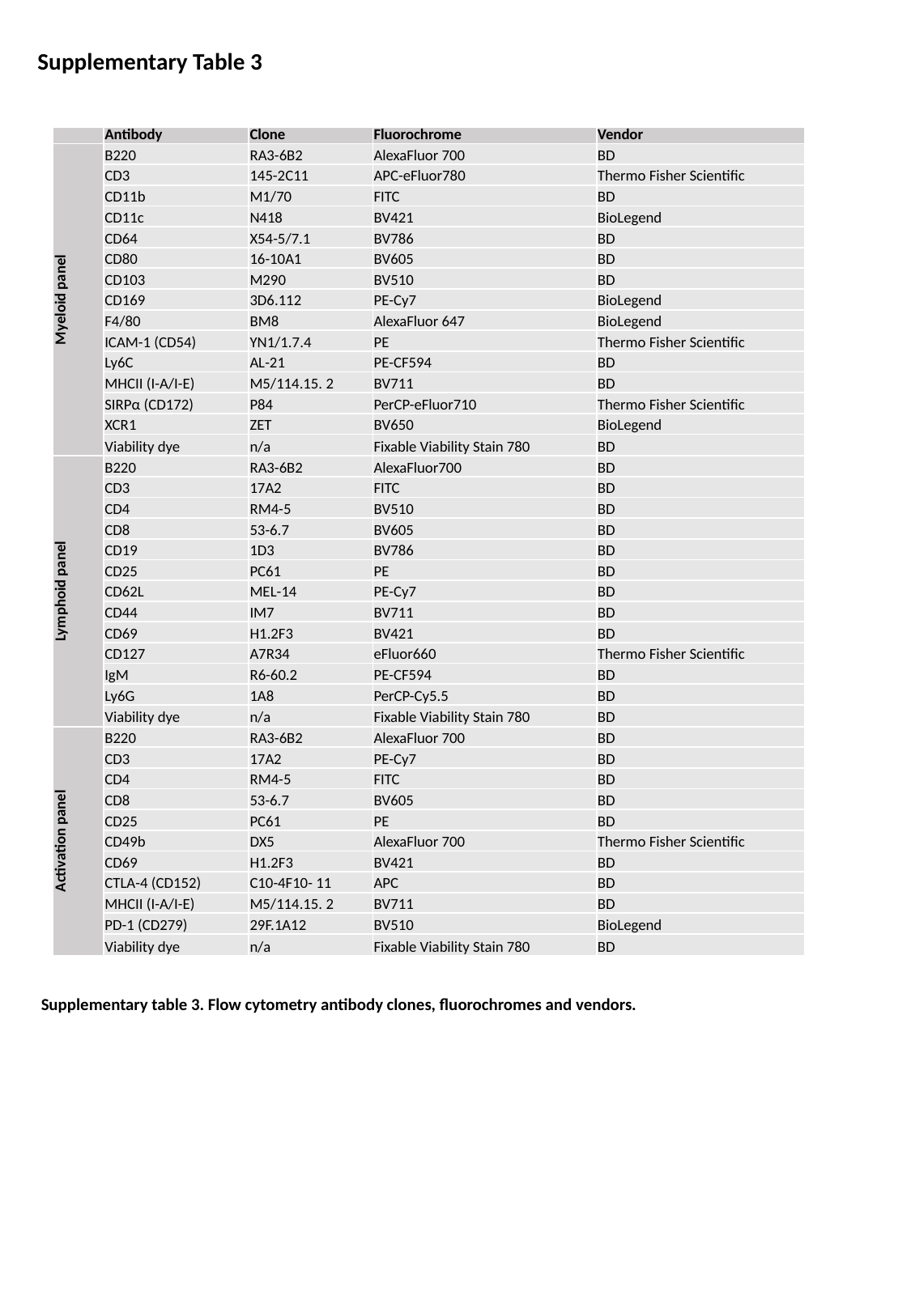

Supplementary Table 3
| | Antibody | Clone | Fluorochrome | Vendor |
| --- | --- | --- | --- | --- |
| Myeloid panel | B220 | RA3-6B2 | AlexaFluor 700 | BD |
| | CD3 | 145-2C11 | APC-eFluor780 | Thermo Fisher Scientific |
| | CD11b | M1/70 | FITC | BD |
| | CD11c | N418 | BV421 | BioLegend |
| | CD64 | X54-5/7.1 | BV786 | BD |
| | CD80 | 16-10A1 | BV605 | BD |
| | CD103 | M290 | BV510 | BD |
| | CD169 | 3D6.112 | PE-Cy7 | BioLegend |
| | F4/80 | BM8 | AlexaFluor 647 | BioLegend |
| | ICAM-1 (CD54) | YN1/1.7.4 | PE | Thermo Fisher Scientific |
| | Ly6C | AL-21 | PE-CF594 | BD |
| | MHCII (I-A/I-E) | M5/114.15. 2 | BV711 | BD |
| | SIRPα (CD172) | P84 | PerCP-eFluor710 | Thermo Fisher Scientific |
| | XCR1 | ZET | BV650 | BioLegend |
| | Viability dye | n/a | Fixable Viability Stain 780 | BD |
| Lymphoid panel | B220 | RA3-6B2 | AlexaFluor700 | BD |
| | CD3 | 17A2 | FITC | BD |
| | CD4 | RM4-5 | BV510 | BD |
| | CD8 | 53-6.7 | BV605 | BD |
| | CD19 | 1D3 | BV786 | BD |
| | CD25 | PC61 | PE | BD |
| | CD62L | MEL-14 | PE-Cy7 | BD |
| | CD44 | IM7 | BV711 | BD |
| | CD69 | H1.2F3 | BV421 | BD |
| | CD127 | A7R34 | eFluor660 | Thermo Fisher Scientific |
| | IgM | R6-60.2 | PE-CF594 | BD |
| | Ly6G | 1A8 | PerCP-Cy5.5 | BD |
| | Viability dye | n/a | Fixable Viability Stain 780 | BD |
| Activation panel | B220 | RA3-6B2 | AlexaFluor 700 | BD |
| | CD3 | 17A2 | PE-Cy7 | BD |
| | CD4 | RM4-5 | FITC | BD |
| | CD8 | 53-6.7 | BV605 | BD |
| | CD25 | PC61 | PE | BD |
| | CD49b | DX5 | AlexaFluor 700 | Thermo Fisher Scientific |
| | CD69 | H1.2F3 | BV421 | BD |
| | CTLA-4 (CD152) | C10-4F10- 11 | APC | BD |
| | MHCII (I-A/I-E) | M5/114.15. 2 | BV711 | BD |
| | PD-1 (CD279) | 29F.1A12 | BV510 | BioLegend |
| | Viability dye | n/a | Fixable Viability Stain 780 | BD |
Supplementary table 3. Flow cytometry antibody clones, fluorochromes and vendors.
